# Checking and replacing fuses

**Published:** 2012

**Authors:** Ismael Cordero

**Affiliations:** Clinical Engineer Email: ismaelcordero@me.com

**Figure:**
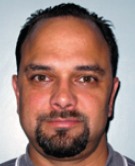
Ismael Cordero

Although we often think of fuses as a nuisance, they play an important safety role in preventing damage to equipment due to electrical overloading, thereby reducing the risk of electrical shock to patients and staff. You should not dismiss a blown fuse as an inconvenience. It may be a sign that a real fault has developed, giving you the chance to find and fix the problem before any serious damage occurs.

Fuses degrade with time and will eventually fail. A blown fuse does not always mean that there is something wrong with the equipment, and in this article we will show you how to replace such a fuse.

However, **do not keep replacing a fuse if it blows immediately after you replace it.** In these instances, call a qualified biomedical equipment technician to service the equipment.

A fuse is essentially a short piece of wire of a selected diameter and composition so that it conducts current up to a certain level, but melts or ‘fuses’ if the current rises above that level. It becomes an open circuit when it blows, interrupting the flow of current and preventing damage.

In most cases, the fuse wire is mounted inside a small glass or ceramic tube, fitted with metal end caps. The glass tube forms a physical guard for the fuse, so that when it blows the molten metal does not cause damage or injury. A glass tube allows you to see when the fuse has blown: there will be a gap in the wire or a metallic smear on the inside of the glass.

## Procedure

Many electrical devices used in eye care have an externally accessible fuse near the electrical cord (Figure 1) that you can check and replace by following these steps.

1. Disconnect the device from the electrical system.
2. Remove the fuse from its holder. In some cases you may need a small screwdriver to unscrew the fuse holder cap.
3. Look at the fuse wire. If there is a visible gap in the wire or a dark or metallic smear inside the glass then the fuse is blown and needs to be replaced. If you cannot see whether the fuse is blown, follow steps 4 and 5. If the fuse is definitely blown, go to step 6.
  **Figure 1.**
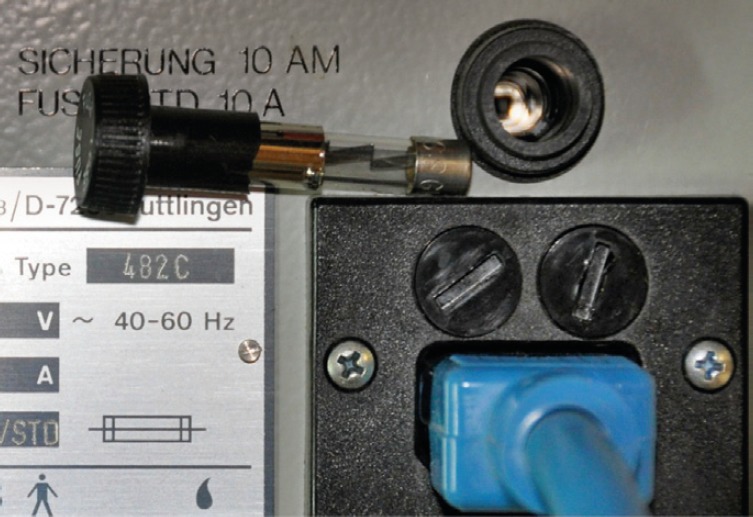

**Figure 2.**
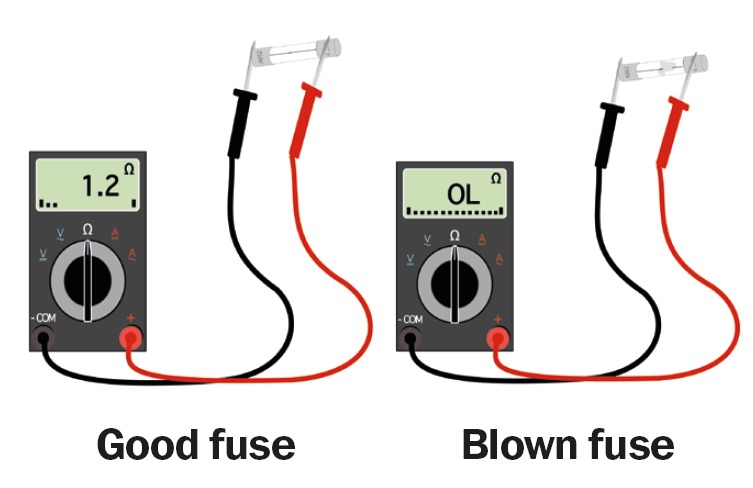
4. Set a multimeter (Figure 2) to the resistance or Ω (Ohms) setting.
5. Place one of the multimeter leads on one end of the fuse. Place the other lead on the other end of the fuse. If the reading is between 0 and 5 Ω (Ohms), the fuse is good. A higher reading indicates a bad or degraded fuse. A reading of OL (Over Limit) definitely means a blown fuse.
6. **If the fuse is blown, replace the fuse with one that is exactly the same** (see panel). Make sure to note the fuse amperage and voltage ratings, which should be marked on the fuse itself (Figure 3) or on the panel label near the fuse holder. Additionally, note the size and whether it is a slow-blow or a fast-blow type fuse. If there are no markings on the fuse itself or on the equipment you must consult the device's operating manual.

## General suggestions

- **Always disconnect equipment from electrical power before removing a fuse; not doing so may result in serious injury.**
- Always replace a fuse with an identical type, and never substitute a fuse with foil or another object. This could lead to electrocution and fires.
- Keep enough stock of the fuses used in your clinic or hospital. Store each type in separate containers with a label describing the fuse's voltage and amperage, whether it is a fast-blow or slow-blow type, the size, minimum number needed (the minimum stock level), and the models of equipment that use each type of fuse.
  **Figure 3.**
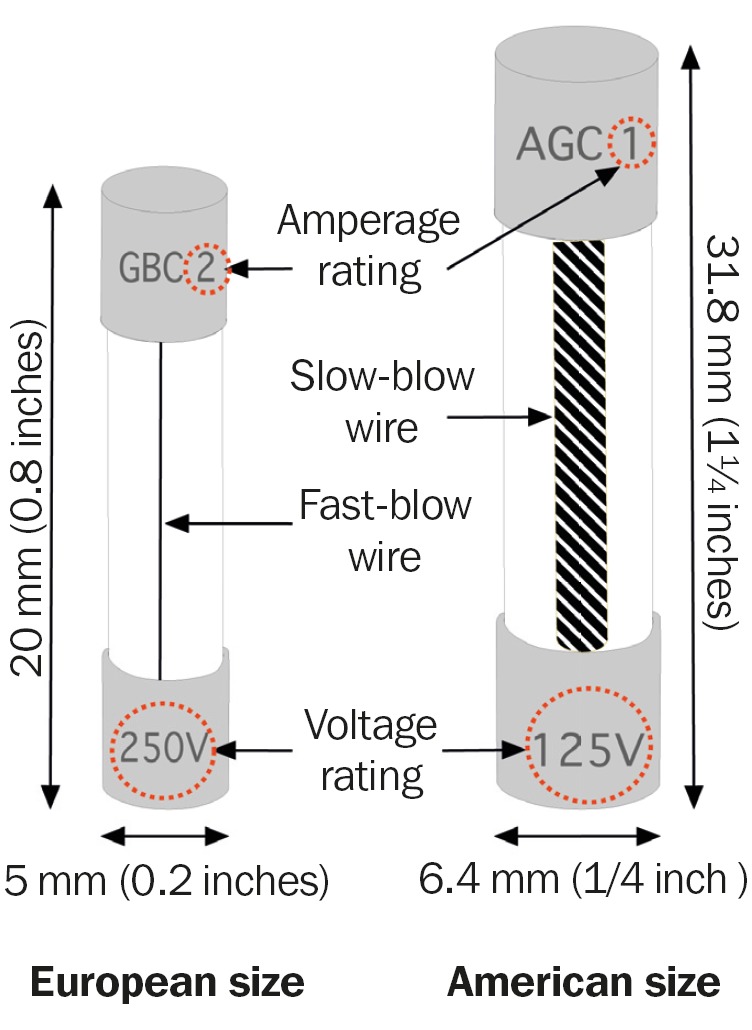
Types of fuses
- Check your stock of fuses frequently and order more fuses when it reaches the minimum stock level.
- If possible, tape a spare fuse to the equipment so that it is available when needed.

**More about fuses**

You should note the following when replacing fuses to ensure an exact match.

**Amperage rating (A).** This indicates how much current the fuse can carry. Most eye care devices have fuses rated between 0 and 10 amperes.

**Voltage rating (V).** This is the maximum supply voltage that the fuse can safely carry. The most popular ratings are 125V and 250V.

**Blowing type. Fast-blow** fuses will blow as soon as the current reaches the fuse's amperage rating, while **slow-blow** fuses are designed to tolerate a large number of startup surges and modest short-term overloads without blowing. Fast-blow fuses usually have a thin wire while slow-blow fuses usually have a thicker, coiled wire. You should never substitute a slow-blow fuse for a fast-blow fuse or vice versa.

**Size and tube material.** Most fuses used in medical equipment have a glass tube but you may find some with ceramic tubes. The two most common sizes of fuses are:

- American size: 3.2 cm × 0.6 cm (¼ inches × ¼ inch),
- European size: 20 mm × 5 mm (0.8 inches × 0.2 inches).

